# Wavelength tunable InGaN/GaN nano-ring LEDs via nano-sphere lithography

**DOI:** 10.1038/srep42962

**Published:** 2017-03-03

**Authors:** Sheng-Wen Wang, Kuo-Bin Hong, Yu-Lin Tsai, Chu-Hsiang Teng, An-Jye Tzou, You-Chen Chu, Po-Tsung Lee, Pei-Cheng Ku, Chien-Chung Lin, Hao-Chung Kuo

**Affiliations:** 1 Department of Photonics & Institute of Electro-Optical Engineering, National Chiao Tung University, Hsinchu 30010, Taiwan; 2 Department of Electrical Engineering and Computer Science, University of Michigan, 1301 Beal Ave., Ann Arbor, Michigan 48109, USA; 3 Institute of Photonic System, National Chiao Tung University, Tainan 711, Taiwan

## Abstract

In this research, nano-ring light-emitting diodes (NRLEDs) with different wall width (120 nm, 80 nm and 40 nm) were fabricated by specialized nano-sphere lithography technology. Through the thinned wall, the effective bandgaps of nano-ring LEDs can be precisely tuned by reducing the strain inside the active region. Photoluminescence (PL) and time-resolved PL measurements indicated the lattice-mismatch induced strain inside the active region was relaxed when the wall width is reduced. Through the simulation, we can understand the strain distribution of active region inside NRLEDs. The simulation results not only revealed the exact distribution of strain but also predicted the trend of wavelength-shifted behavior of NRLEDs. Finally, the NRLEDs devices with four-color emission on the same wafer were demonstrated.

GaN-based LEDs have been widely used in our daily life, such as light communication, lighting and display. However, the efficiency still suffers from the poor internal quantum efficiency (IQE) and low light extraction efficiency (LEE). The quantum confined Stark effect (QCSE), which rises from built-in field in the strained wurtzite materials and leads to spatial separation of electrons and holes, can seriously deteriorate the IQE of the GaN-based multiple quantum well devices^1^,^2^,^3^,^4^,^5^. Therefore, eliminating the QCSE becomes an important issue for high efficiency applications. Many method, such as GaN growth on the semi-polar or non-polar crystal planes, can effectively solve the problem, however, this important can come with a high price tag and much difficult growth parameters to be tuned^6^,^7^,^8^,^9^.

Nano-LEDs based on InGaN/GaN multiple quantum wells (MQWs) has been identified as a viable solution to unravel long pending issues in solid state lighting such as QCSE, inefficient light extraction and efficiency droop^10^,^11^,^12^. Especially for QCSE, any modification in the internal field and strain can affect its magnitude, and thus effective bandgap^13^,^14^. The tiny sizes of the nano-LEDs set up a perfect condition for such change and, it should be noted that the level of strain can be managed by the size of nano-LEDs due to the relaxation brought by tiny volume with lattice mismatch. Consequently, the emission wavelength can be tuned by adjusting the surface to volume ratio of nano-LEDs due to the different level of screening of QCSE^15^,^16^. Based on this feature, multi-color emission on a LED wafer can be realized by fabricating the different sizes of a nano-structure. Most of previous studies reported the nano-rod LEDs with strain relaxation and improved light extraction efficiency (LEE)^17^,^18^,^19^. However, controlling the conventional nano-rod LED via nano-sphere lithography is not easy. First, we have to overcome the non-uniform distribution of the nano-sphere, especially for the smaller diameter sizes (<200 nm)^20^. Second, the nanorod could have very different strain distribution because of the localized difference between the center and the edge^21^,^22^.

In this study, nano-ring LEDs were fabricated by nano-sphere lithography. The improved IQE was achieved by the reduced QCSE due to nano-scaled strain relaxation. Importantly, the effective bandgap of nano-ring LEDs can be precisely tuned through modifying the wall width during the fabrication process. Consequently, the emission wavelength tuning capability of nano-ring LEDs through strain engineering can be realized, the color of LEDs can be tuned from green to blue (535 nm to 480 nm). This result presents the possibility to obtain the different color LEDs on one LED epitaxial wafer, which can be utilized to micro display pixel and multi-channel visible light communication (VLC) system.

## Results

**Figure 1:**
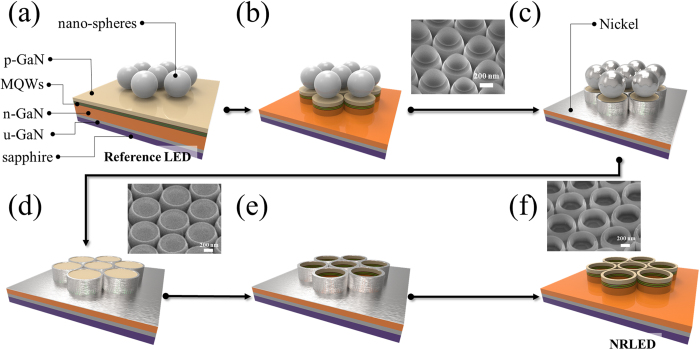
Process flow of nano-ring LEDs. (**a**) Spin coating polystyrene (PS) nano-spheres homogeneously on the Reference LED wafers. (**b**) Generated nano-rods arrays through the ICP-RIE process. The insert presents SEM image of nano-rods LED. (**c**) Reducing the diameter of nano-spheres via oxygen plasma treatment and depositing the nickel metal on nano-rods LED with nano-spheres. (**d**) Nano-rods LED with the nickel metal as protected layer. The insert presents SEM image of nano-rods LED with nickel. (**e**) After the ICP-RIE etching process, nano-ring LEDs template were produced. (**f**) Removing the nickel by acid solution, the nano-ring LEDs was produced. The insert presents SEM image of nano-rings LED.

The GaN-based LED was grown on a *c*-plane sapphire substrate by metal–organic chemical vapor deposition (MOCVD). Depositing low-temperature GaN nucleation layer of 30 nm, a 2 μm-thick u-GaN layer as a buffer layer, and followed by 7 periods of In_0.28_Ga_0.72_N/GaN (3 nm/10 nm) MQWs sandwiched by a 2 μm-thick Si-doped n-GaN layer and a 120 nm Mg-doped p-GaN layer. After growing the InGaN/GaN MQW LEDs, the nano-ring process was applied to achieve the strain management in InGaN/GaN MQW. We applied nano-sphere lithography to fabricate the nano-ring structure in InGaN MQW LEDs, which exhibits the advantages of low cost, large-area fabrication, controllable wall width and to tune emission wavelength of nano-rings. First, spin coating polystyrene (PS) nano-spheres whose diameter is approximately 900 nm on the GaN LED epitaxy surface, as shown in Fig. 1a. Then, inductively coupled plasma reactive ion etching (ICP-RIE) was utilized to etch the GaN-based material, forming a nano-rod array with residual nano-spheres, as shown in Fig. 1(b). Next, the diameter of the nano-spheres can be reduced via oxygen plasma treatment, and through this step, it is possible to control the final wall width of the nano-ring. Using the electron beam evaporator system, the nickel (Ni) metal was deposited on the nano-rod with the residual nano-spheres, as shown in Fig. 1c. Ultrasonic cleaning machine was applied to remove the nano-spheres, as shown in Fig. 1d. The residual Ni can protect a part of nano-rod during the second etching process and further form the nano-ring structure, as shown in Fig. 1e. Finally, the Ni was removed by HCl solution and the complete nano-ring template was achieved, as shown in Fig. 1f. The GaN LED epitaxy wafer without the nano-ring process is Reference LED. After the nano-ring process, three difference wall widths NRLED as compared with Reference LED. The Reference LED sample has the same epitaxial structure as the NRLEDs, but only go through the standard LED process.

Starting the bare nano-ring wafers (Fig. S1a), in order to avoid the short circuit happened, depositing 200 nm-thick SiO_2_ by plasma-enhanced chemical vapor deposition (PECVD) on the nano-ring LED as preservation layer to isolate p-and n-GaN layer (Fig. S1b). Then, photoresist was coated to fill the gap between nano-ring LEDs and the center of nano-ring LEDs. Its thickness must be over the height of nano-ring (Fig. S1c). Afterward, an etch-back treatment was applied to the photoresist layer until the SiO_2_ at the top of nano-ring structure was revealed, using ICP-RIE to etch the top SiO_2_ of nano-ring until the p-GaN was revealed (Fig. S1d). Following above fabrication method, the nano-ring LED template with revealing p-GaN (Fig. S1e) was made. Finally, the Indium Tin Oxide (ITO) was deposited (Fig. S1f), the standard LED wafer can be produced and we can follow the face-up standard process of LED to make the LED device with Ni/Au (10 nm/50 nm) metal as the electrode.

**Figure 2:**
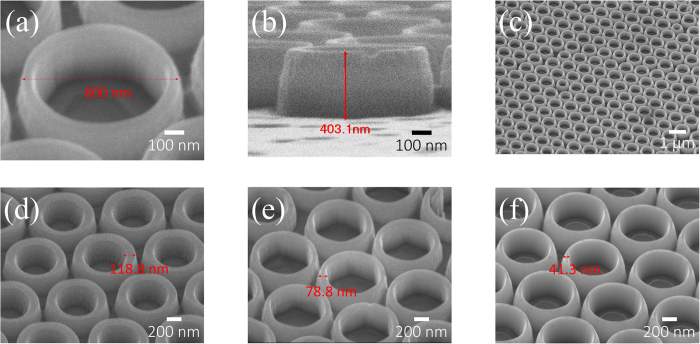
Scanning electron microscope image of nano-ring LEDs. Every single ring, (**a**) the outside diameter and (**b**) the height of a nano-ring is approximately 800 nm and 400 nm, respectively. (**c**) In this approach, it was able to create a larger nano-ring array. Besides, to compare wavelength-shifting behavior, we altered different wall widths of nano-ring which is (**d**) 120 nm, (**e**) 80 nm and (**f**) 40 nm.

To investigate the characteristics of nano-rings LED, we fabricated three nano-ring LEDs with fixed the outside diameter and height in about 800 nm and 400 nm, as shown in Fig. 2a,b. Our approach is powerful to create large nano-rings array on the LED wafer, as shown in Fig. 2c. The wall width we fabricated 120 nm (Fig. 2d), 80 nm (Fig. 2e) and 40 nm (Fig. 2f). Through high-resolution transmission electron microscopy (HRTEM), to confirm our active region is still complete after the nano-ring process, as shown on Fig. S2. The interface of MQWs is smooth and the quality of epitaxy is very good. In addition, after the nano-ring process, the edge of active region is still sharp, and without dislocations and pits.

**Figure 3:**
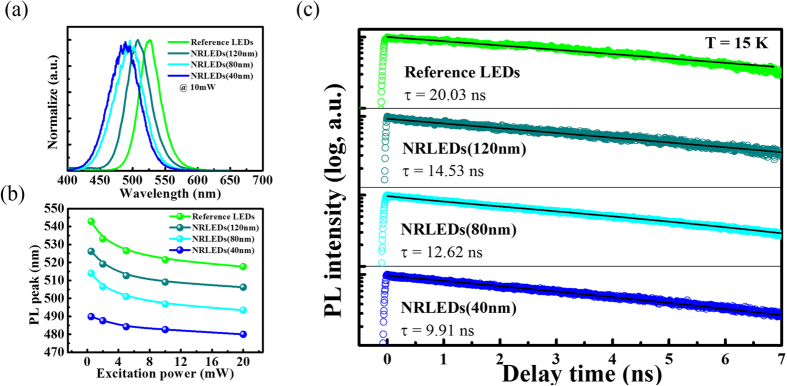
Spectral analysis. (**a**) The PL spectrum with excitation power of 10 mW. (**b**) PL emission peak shifting of nano-ring LED with different wall widths as a function of excitation power. The magnitude of blue-shifted of Reference LEDs, NRLEDs (120 nm), NRLEDs (80 nm) and NRLEDs (40 nm) is 25.2 nm, 19.8 nm, 20.5 nm and 9.9 nm, respectively. (**c**) TRPL measurement and the fitting curves for Reference LEDs and NRLEDs.

The active region consisted of InGaN and GaN thin film. Therefore, there is a larger strain inside the active region because of the lattice mismatch of InGaN and GaN. Piezoelectric field-induced QCSE can lead to the significant blue-shifting of photoluminescence (PL) emission peak on power-dependent measurement due to the stronger screening effect under the higher carrier density in the active region^23^ and reduce the hole and electron wave-functions overlap^24^, which would increase the radiative recombination time. Figure 3 presents the PL spectrum of each sample. There is a significant blue-shifting of emission peak with deceasing the wall width of nano-ring LEDs from 120 nm to 40 nm under the excitation power of 10 mW, as shown in Fig. 3a. Compared to previously nano-rod-typed studies^22^, the nano-ring structure can maintain highly monochromatic spectrum since the nano-ring structure has a more uniform strain distribution in the active region (we will discuss below), the full width at half maximum (FWHM) of Reference LEDs, 120 nm, 80 nm and 40 nm (NRLEDs) were 37 nm, 42 nm, 52 nm and 51 nm, respectively. From our previously study^25^, the increase of carrier in the active region can lead to the screening of the built-in field existing in the device. This screen can reduce the QCSE and move the emission wavelength towards shorter side^26^. So the amount of this blue-shift can be an indicator of the original magnitude of the built-in field. The internal strain in the device is the direct source of the QCSE and thus we can correlate the internal strain with the amount of the blue-shift in our experiment. As shown in Fig. 3b, the amount of blue-shift drops as the width drops, and thus the 40 nm device has a much less strain inside the ring compared to the 120 nm case. According to coulomb screening effect theory, we can know the QCSE of NRLEDs are exactly smaller than Reference LEDs, moreover, the NRLEDs with the wall width of 40 nm has the smallest blue-shifting behavior. The internal quantum efficiency (IQE) behavior also be improved as decreased the wall width, as shown in Fig. S5 (see Supplementary Note 1).

Additionally, the strain relaxation process would decrease the radiative recombination time due to improving hole and electron wave-functions overlap and further increasing radiative recombination rate. Time-resolved PL (TRPL) measurements performed at 300 K for ensembles of nano-rings. Figure 3c shows the PL decay time at the PL emission peak of each sample. The radiative recombination lifetime of LEDs were 14.28 ns, 9.8 ns, 6.87 ns and 5.49 ns for Reference LEDs, NRLEDs (120 nm), NRLEDs (80 nm) and NRLEDs (40 nm) indicated that the radiative recombination rates are enhanced by factor of 1.5, 2.1, and 2.6, respectively, the NRLEDs have the faster decay time, which mean the NRLEDs have a larger hole and electron wave-functions overlap than Reference LEDs^27^,^28^. On a planar active region situation, the piezoelectric field-induced strain affect will result in band-titled phenomenon in the active region.

**Figure 4:**
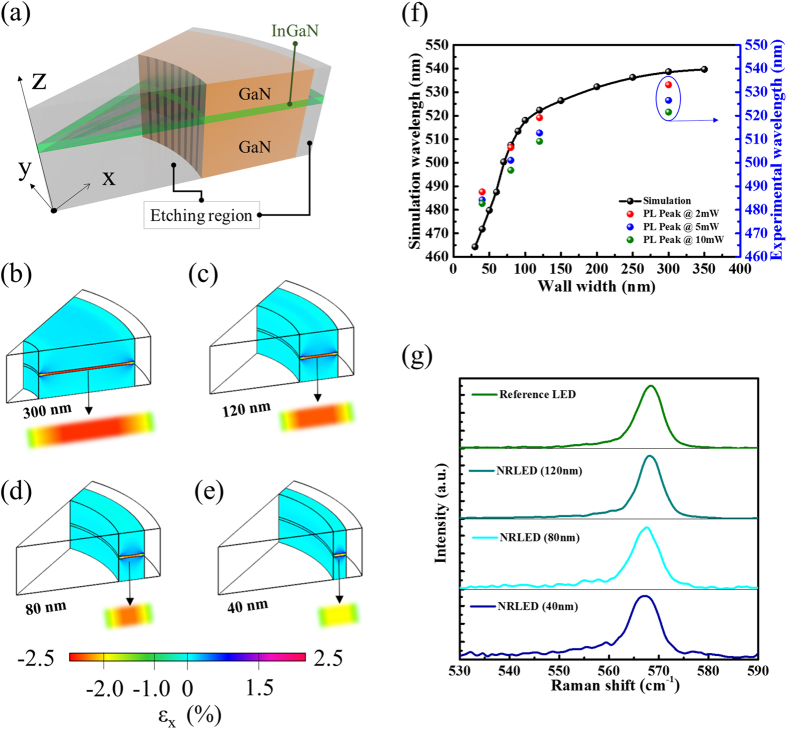
Simulating the strain variation of nano-ring active region with decreasing wall width of a ring. The strain distribution of active region of (**b**) Reference LEDs and the nano-ring with (**c**) 120 nm (**d**) 80 nm and (**e**) 40 nm wall width. (**f**) The wavelength-shifting behavior trend of simulated and experimental results. (**g**) Raman spectra of Reference LED and NRLED with different wall widths.

From our spectral analysis, the strain released process can suppress the QCSE and cause a significant blue-shift of PL emission peak. Therefore, in order to further understand the strain distribution of active region inside nano-ring LEDs, we simulated the strain relaxation with the wall width reduction of a nano-ring structure by the finite elemental method. To follow the actual etching process, we enlarge the inner circle of a nano-ring step by step but fixed the outer diameter, as shown in Fig. 4a. In this simple model, a 3-nm-thick In_0.28_Ga_0.72_N was sandwiched in between two GaN barriers. The strain tensors ε_x_ at the cross section of x−z plane of nano-ring LEDs with wall width of 300 nm (Fig. 4b), 120 nm (Fig. 4c), 80 nm (Fig. 4d) and 40 nm (Fig. 4e) were calculated. The strain tensors on both outer and inner peripheral areas show considerable relaxations at the sidewall of a nano-ring structure. With decreasing the wall width of a nano-ring, the strain magnitude in the central region (Black dash line in Fig. S3a) was reduced from −2.3% to −1.7% (Fig. S3b). Therefore, the better strain-relaxation characteristic of nano-ring structures can be expected as compared to the nano-rods structure with the same diameter (Fig. S3a,b) and the strain magnitude in active region of nano-rod and nano-ring is −2.4% and −1.7%, respectively (Fig. S4d). In a third case, a nano-scale rod with the same diameter (40 nm) compared to the width of the nano-ring was set up for strain calculation. In this case, the relaxation of the strain in the active region is similar to the nano-ring case (as shown in Fig. S4c,d). As a short summary on these simulations, we found that the rod with the same outer diameter as the nano-ring definitely bear higher strain after the etch process due to solid nature of the structure which can preserve the difference between lattice constants of different materials. Meanwhile much less materials are left in the nano-ring case, and this can facilitate the strain relaxation just like the nano-rod with similar scale of material in radial direction. It can be clearly observed that the strain tensor in the whole InGaN quantum well were reduced as the wall width of nano-ring deceases, which is attributed to the ratio of strain-relaxed active region to the strained region size becomes larger as the diameter decreases. With the reduced strain tensors, the suppression of QCSE can be expected. Corresponding to experimental results, the trend of both results are similar, as shown in Fig. 4f. In Fig. 4g, the Raman peaks shift toward a lower value, the strain is relieved in active region. Compared to the E_2_ phonon peak of strain-free GaN (566.5 cm^−1^), 1.99 and 0.73 cm^−1^ of the Raman peak shift correspond to 0.89 and 0.32 GPa of stress for the Reference LED and NRLED with 40 nm wall width, respectively. According to Hooke’s law, the stress is positive correlation with strain so the strain tendency of experiment is similar to our simulation result, as shown in Table 1 (see Supplementary Note 2). From the simulation results, the blue-shifting behavior is not a linear tendency and it has a reverse point which appear on roughly 100 nm. The strain relaxation here would slow down. That can explain why the blue-shifting of our PL experimental results on 120 nm and 80 nm is almost the same in Fig. 3b.

**Figure 5:**
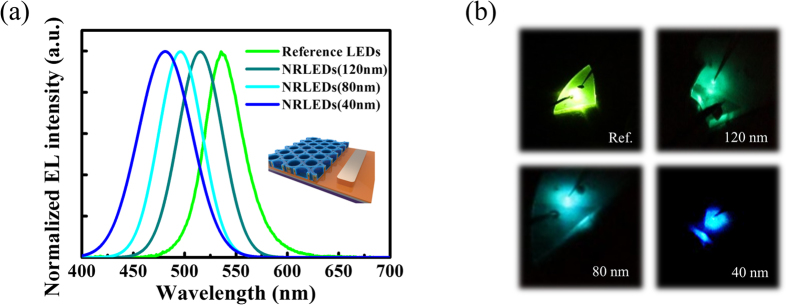
Electroluminescence (EL) characterization. (**a**) The EL spectrum and (**b**) real photograph of nano-rings LED in different wall widths.

Figure 5a shows the electroluminescence (EL) spectrum of reference LED and the NRLEDs with the wall width of 120 nm, 80 nm and 40 nm. The EL spectrum of nana-ring LEDs would have a significant blue-shift as the wall width of the ring reduces. The measured widest shift is 55 nm. The emission wavelength tuning capability of nano-ring LEDs through strain engineering can be clearly observed and the color of LEDs can be tuned from green to blue (535 nm to 480 nm), as shown in Fig. 5b. This result presents the possibility to obtain the LEDs with different emission colors on one LED epitaxial wafer, which can be applied to micro or nano-display pixel and multi-channel visible light communication (VLC) system.

## Conclusion

In summary, we demonstrated how to fabricate the nano-ring structure with three kinds of wall widths of 120 nm, 80 nm and 40 nm. According to spectral analysis, we discovered the emission wavelength of nano-ring LEDs have a significant shift from green to blue and the magnitude of blue-shifted was increased with decreasing the wall width of nano-ring LEDs. Furthermore, via the TRPL measurement, when wall width of a nano-ring is decreased, the carrier radiative recombination lifetime time was decreased due to the suppressed QCSE and increasing hole and electron wave-functions overlap. Through the simulation of strain distribution, the results showed the strain in the active region is relaxed. That means the reducing wall width can powerfully suppress the QCSE and modify the emission wavelength of nano-ring LEDs. We believe this nano-ring LED design can provide a feasible solution for the future realization of monolithic integration of RGB LEDs in nano-meter scale.

## Methods

### Growth GaN-based materials

First, the p-type dopant in this device is Mg and carried into growth chamber by Cp_2_Mg precursor. As we finished the growth of p-GaN, a 20 minutes of post-growth annealing will be carried out the dopant activation. The post growth annealing was kept at 650 °C and only in N_2_ ambient until the dopant was activated. The doping level is [Si] =1 × 10^19^ cm^−3^ for n-GaN and [Mg] = 1 × 10^19^ cm^−3^ for p-GaN, respectively. The Si dopant can be nearly 100% activated but only 3% of Mg can be activated, which means that the hole concentration is only 3 × 10^17^ cm^−3^.

### ICP-RIE

A planar type ICP-RIE system is SAMCO ICPRIE 101iPH. The ICP and bias power source with RF frequency of 13.56 MHz. The ICP system has reactor and load-lock chambers. The reaction gases of Cl_2_ and Ar were introduced into reactor chamber through independent electronic mass flow controllers (MFCs) that can precisely control the gas flow rate of each gas with about 1 standard cubic centimeter min (sccm). The etching rate is approximately 6000 Å/min and a gas mixture condition of Cl_2_ and Ar is 50 and 20 sccm, respectively. During the etching process, the ICP and bias power were maintained at 200 W with chamber pressure of 0.33 Pa.

### EL measurement

The light of the sample can be collected by a fiber. The power supplies (Keithley 2400) can supply different currents to the sample and the spectrometer can analyze the intensity of each wavelength in the sphere.

### Numerical simulation

The electromechanical equations of nitride-based piezoelectric materials included both the direct and the reverse piezoelectric effects. We adopt the finite element method to analyze the electromechanical equations^29^. Since the optical properties of InGaN/GaN nanostructures depend heavily on the strain field and the electric potential in and around MQWs, the single particle state energies of electron and hole were calculated by using the four-band k·p Hamiltonian which includes the coupling tween the conduction band and three valence bands^29^.

## Additional Information

**How to cite this article**: Wang, S.-W. *et al*. Wavelength tunable InGaN/GaN nano-ring LEDs via nano-sphere lithography. *Sci. Rep.* **7**, 42962; doi: 10.1038/srep42962 (2017).

**Publisher's note:** Springer Nature remains neutral with regard to jurisdictional claims in published maps and institutional affiliations.

## Supplementary Material

Supplementary Information

## Figures and Tables

**Table 1:** Raman shift and stress of Reference LED and NRLEDs.

	Reference LED	NR LED (120 nm)	NRLED (80 nm)	NRLED (40 nm)
Raman peak (cm^−1^)	568.49	568.10	567.42	567.23
Stress (Gpa)	0.89	0.71	0.41	0.32
