# Supplementary material for: Wavelength tunable InGaN/GaN nano-ring LEDs via nano-sphere lithography
Source: Sci Rep. 2017 Mar 3;7:42962. doi: 10.1038/srep42962 (PMC5335604; doi:10.1038/srep42962)
Supplement: Supplementary Information [file srep42962-s1.pdf]

Supplementary Information

Wavelength tunable InGaN/GaN nano-ring LEDs via nano-sphere lithography

Sheng-Wen Wang[1], Kuo-Bin Hong[1], Chu-Hsiang Teng[3], Yu-Lin Tsai[1], An-Jye Tzou[1], You-Chen Chu[1], Po-Tsung Lee[1], Pei-Cheng Ku[3], Chien-Chung Lin[2,†] and Hao-Chung Kuo[1,*]

[1]Department of Photonics & Institute of Electro-Optical Engineering, National Chiao Tung University, Hsinchu 30010, Taiwan.
[2]Institute of Photonic System, National Chiao Tung University, Tainan 711, Taiwan.
[3]Department of Electrical Engineering and Computer Science, University of Michigan, 1301 Beal Ave., Ann Arbor, Michigan 48109, USA.

[†]chienchunglin@faculty.nctu.edu.tw; [*]hckuo@faculty.nctu.edu.tw

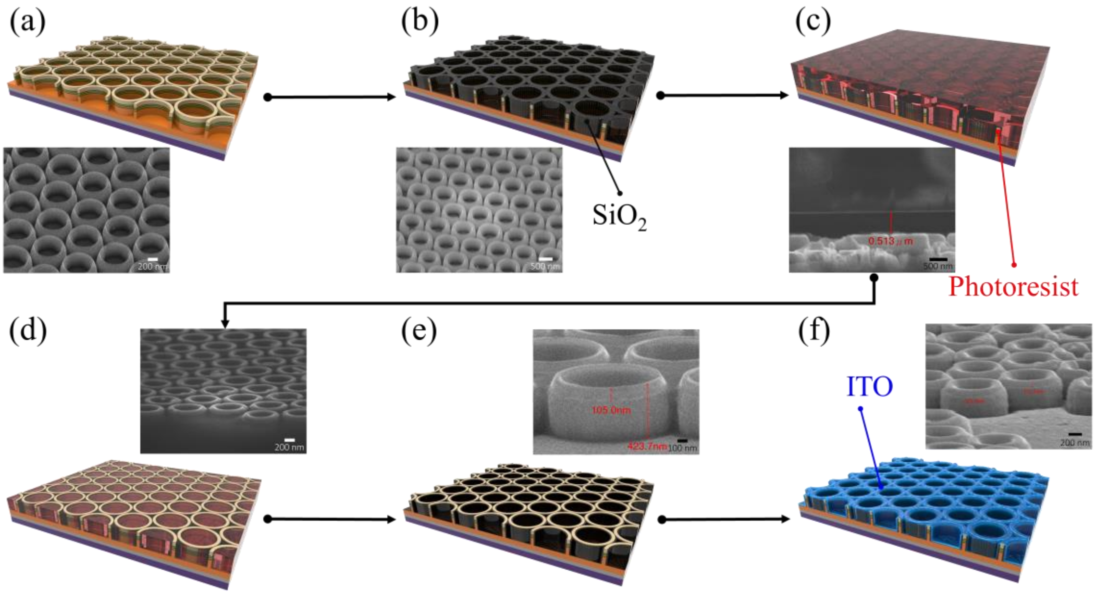

Supplementary Figure S1. Devices process follow. (a) Preparing a nano-rings LED. The insert presents SEM image of bare nano-rings LED. (b) To avoid the short circuit issue, depositing SiO_2 as preservation layer on bare nano-rods LED everywhere. The insert presents SEM image of bare nano-rings LED with SiO_2. (c) Spin coating photoresist whose thickness must be over the height of a nano-ring on the nano-rings LED with SiO_2. The insert presents SEM image of bare nano-rings LED with SiO_2. (d) Using RIE system etch the photoresist and SiO_2 until revealing the p-GaN, as inserted SEM image. (e) Removing the photoresist, nano-rings LED with preservation layer and bare p-GaN can be made. The insert presents SEM image of nano-rings LED with revealing p-GaN. (f) Directly depositing ITO film on the nano-rings LED, the ITO is able to connect the p-GaN layer of nano-rings LED. The insert presents SEM image of nano-rings LED with ITO layer.

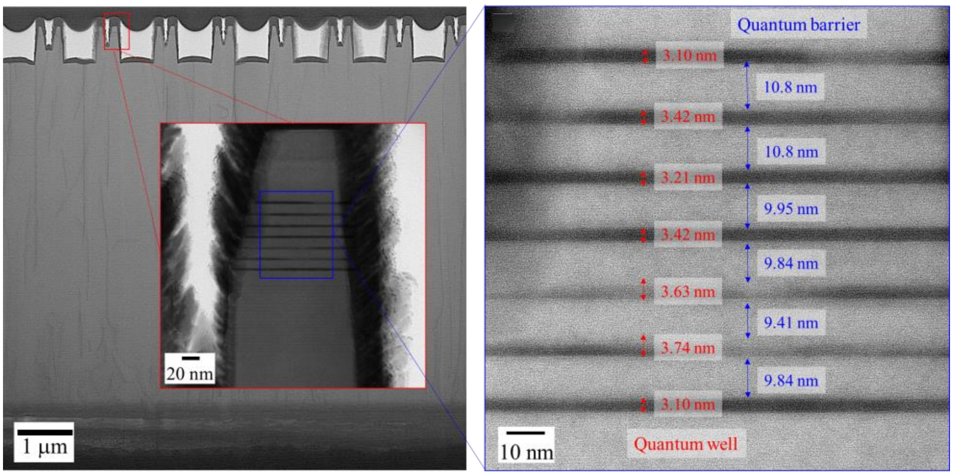

Supplementary Figure S2. HRTEM image of NRLED with 80 nm wall width.

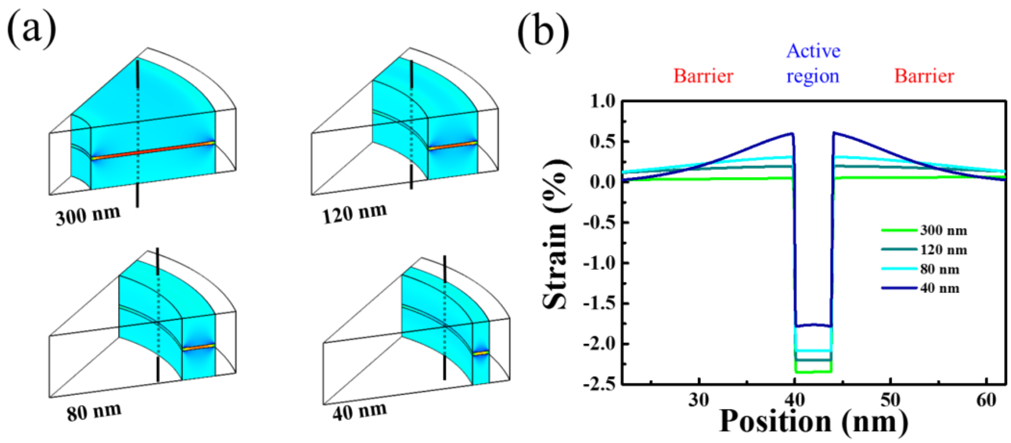

Supplementary Figure S3. Simulating the strain variation of ring structures. The strain distribution of active region of (a) nano-ring with 300, 120, 80 and 40 nm in wall width. For compression, (b) shows the magnitude of strain in the center (black dash line) of nano-ring.

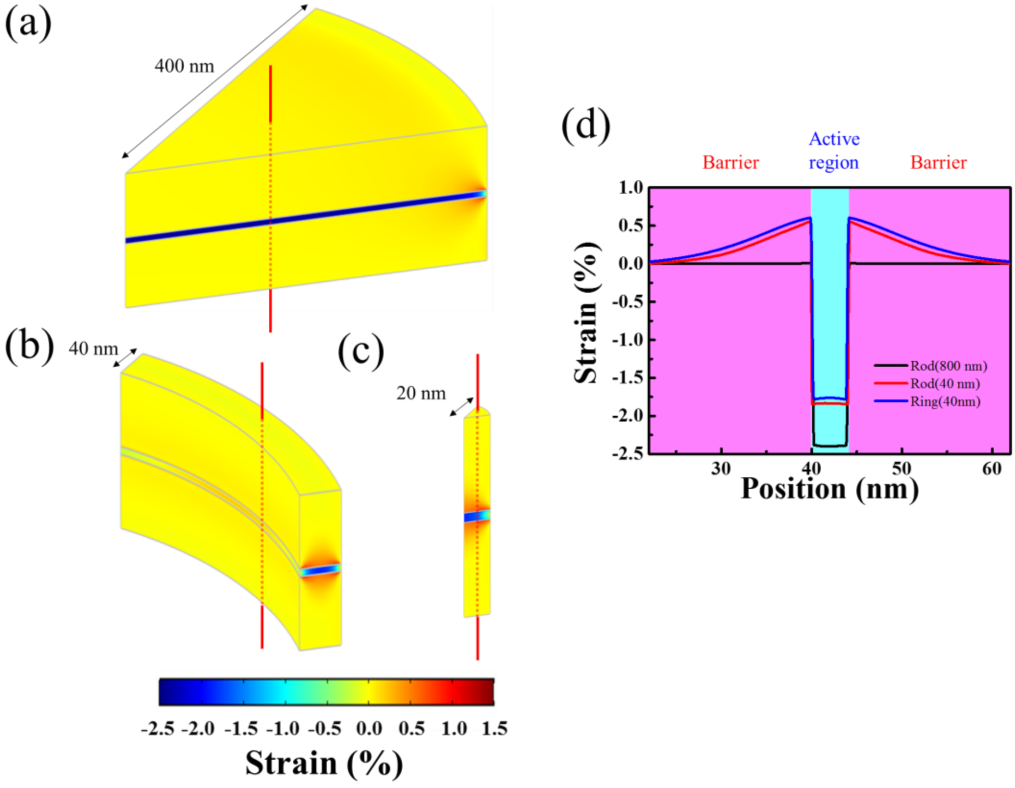

Supplementary Figure S4. Simulating the strain variation of ring and rod structures. The strain distribution of active region of (a) nano-rod with 800 nm in diameter, (b) nano-ring with 800 nm in diameter and 40 nm in wall width and (c) nano-rod with 40 nm in diameter. For compression, (d) shows the magnitude of strain in the center (red dash line) of nano-ring (with 40 nm wall width) and nano-rod (with 40 nm diameter).

Supplementary Note 1: The internal quantum efficiency (IQE) behavior of NRLEDs with different wall width

In InGaN-based MQWs, the band structure of quantum well has a serious bending phenomenon cause decreased the overlap of electron and hole wave functions and further affected the lower internal quantum efficiency (IQE)[1]. According to equation (1)[2], the injected carrier density can be determined by the pumping power of a laser (P), the energy of photon ($h\nu$), the beam size of a laser (ϕ), the thickness of active region (d_{active}), quantum barrier (d_{GaN}) and well (d_{InGaN}), the repetition rate of a laser (f), the absorption coefficient of GaN (α_{GaN}) and InGaN (α_{InGaN}), and the reflectance of pumping laser (R).

$$\text{Injected Carrier Density} = \frac{P}{(h\nu)\times\phi\times d_{active}\times f}\times\exp(-\alpha_{GaN}d_{GaN})\times\left[1-(-\alpha_{InGaN}d_{InGaN})\right]\times(1-R) \qquad (1)$$

Based on our power-depended PL and equation (1), we can estimate the internal quantum efficiency as function of injected carrier density and further observe the droop behavior of each sample. Fig. 2 shows the IQE of each sample, the droop behavior of the NRLEDs were better than Reference LED. The reason of different current densities in each sample is using the same function of excitation power. After the calculation, the injection current density will be dependent on the area of active region so the smallest active region has the highest injection current density.

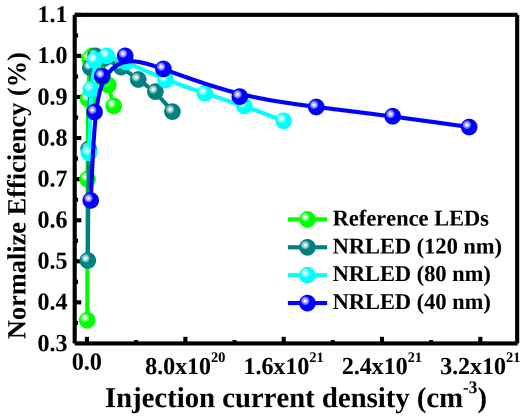

Supplementary Figure S5. IQE behaviour of Reference LED and NRLEDs with different wall widths.

Supplementary Note 2: The Raman spectra and stress of NRLEDs with different wall width

The strain of GaN epitaxial layers can be estimated by the shift of the E_2 mode. In Fig. 2, the E_2 phonon peaks of Raman scattering were observed at 568.49, 568.10, 567.42 and 567.23 cm^{-1} for Reference LED, NRLED with 120, 80 and 40 nm wall width, respectively. As the Raman peaks shift toward a lower value, the strain is relieved in active region. The relation between the stress and the Raman peak shift of E_2 mode can be calculated from equation (1):

$$\Delta\omega_{E_2} = \omega_{E_2} - \omega_0 = C\sigma \quad (1)$$

where the is ω_{E_2} the measured E_2 phonon frequencies, ω_0 is phonon frequencies of

Reference

1 DenBaars, S. P. *et al.* Development of gallium-nitride-based light-emitting diodes (LEDs) and laser diodes for energy-efficient lighting and displays. *Acta Mater.* **61**, 945-951 (2013).

2 Chiu, C. H. *et al.* High efficiency GaN-based light-emitting diodes with embedded air voids/SiO2 nanomasks. *Nanotechnology* **23**, 045303 (2012).
